# Antibacterial and antioxidant activity of Portuguese *Lavandula luisieri* (Rozeira) Rivas-Martinez and its relation with their chemical composition

**DOI:** 10.1186/s40064-016-3415-7

**Published:** 2016-10-04

**Authors:** Sofia Pombal, Cleide F. Rodrigues, João P. Araújo, Pedro M. Rocha, Jesus M. Rodilla, David Diez, Ángela P. Granja, Arlindo C. Gomes, Lúcia A. Silva

**Affiliations:** 1 FibEnTech- Materiais Fibrosos e Tecnologias Ambientais, Departamento de Química, Universidade da Beira Interior, Rua Marquês d’Ávila e Bolama, 6201-001 Covilhã, Portugal; 2 Departamento de Química Orgánica, Facultad de Ciencias Químicas, University of Salamanca, Plaza los Caidos s/n, 37008 Salamanca, Spain

**Keywords:** *Lavandula luisieri*, Oil composition, Phenol content, Flavonoid content, Antioxidant activity, Antibacterial activity

## Abstract

*Lavandula luisieri* (Rozeira) Rivas-Martinez is an endemic aromatic *Labiatae* the Iberian Peninsula, common in semi-arid regions of southern Portugal and southwestern Spain, that produces an active antibacterial essential oil from the leaves and flowers. This work presents the study of the chemical variation in various stages of growth of leaves and flowers of *L. luisieri.* It has been found that the essential oils are mainly constituted by 1,8-cineol, camphor, linalool and trans-α-necrodil acetate. It was also studied the total phenol content and the antioxidant activity on leaves and flowers. The ethanol extraction from de leaves contents the highest total phenol, important factor for the antioxidant activity of the plant, extract. It has been studied too, the antibacterial activity against *Escherichia coli, Salmonella* spp*. and Staphylococcus aureus*. In accordance with the obtained results, the antibacterial activities stand out against *Staphylococcus*, of the oil of *L. luisieri* (leaves and flowers).

## Background

*Lavandula luisieri* (Rozeira) Rivas-Martinez is an aromatic *Labiatae* endemic to the Iberian Peninsula, common in the semi-arid regions of Southern Portugal and Southwest Spain. Essential oils of *Lavandula* species are of economic value for the fragrance industry (Lavoine-Hanneguelle and Casabianca 2004).

*Lavandula* species have been proved to possess antispasmodic actions on both intestinal (ileum) and uterine smooth muscle in animal studies (Cavanaghan and Wilkinson 2002). Lavender oils have also been reported to possess biological activity against many species of bacteria and fungi, as well as acaricidal activity. In dermatology lavender oil can be used in wound healing and for relieving the symptoms of psoriasis, dermatitis and eczema (Cavanaghan and Wilkinson 2002) and as a potential natural biopesticide (Matos et al. 2009).

Extensive research has been dedicated to identification of antioxidant compounds of natural sources, among them phenolic compounds and their possible use in processed foods as a natural antioxidant have reached a new high in recent years.

Studies with the essential oils of *L. luisieri* collected in Portugal show that is an oil presents a wide variation in their chemical composition and yield obtained oil; Their major components were fenchone, camphor, trans-α-necrodol, trans-α-cecrodyl acetate and β-selinene (González-Coloma et al. 2011). Comparison of samples from Portugal and Spain (Toledo, Seville) detect differences in chemical composition (González-Coloma et al. 2006). In this study were found as major components: camphor, 1,8-cineole, 2,3,4,4-tetramethyl-5-methylen-2-cyclopenten-1-one. These oils are analyzed by GC–MS and their antifeedant effects tested against several insect species, the aphid *Myzus persicae* being the most sensitive.

The main objective of this work is to determine the essential oil composition of flowers and leaves of *L. luisieri* of a specific place in Portugal, subsequent analysis of phenols and flavonoids content and antibacterial activity studies using two different techniques: diffusion agar with cylindrical cavities and vapour diffusion teste in agar plate.

## Results and discussion

### Chemical composition analysis

*Lavandula luisieri* essential oils are very distinct from other *Lavandula* species. Although significant quantitative differences may occur in the amounts of the main compounds, irregular monoterpenes are always present.

Many studies have been published about the chemical composition of essential oil of *Lavandula stoechas* L., but very few report the essential oil of the study *L. luisieri*.

Some studies have shown that *L. luisieri* has an atypical composition, giving it unique characteristics in the plant kingdom and therefore can be considered a chemotaxonomic marker of this species. Garcia-Vallejo (1992) was the first to identify these compounds. Later on, different authors found other uncommon monoterpenes, hence confirming the peculiarity of *L. luisieri* essential oils (Baldovini et al. 2005; González-Coloma et al. 2011; Lavoine-Hanneguelle and Casabianca 2004; Sanz et al. 2004). It was found, along 1,8-cineole, lavandulol, linalool and their acetates, a variety of compounds with a structure of 1,2,2,3,4-pentamethylcyclopentane (necrodane) such as α-necrodol acetate and α-necrodile (Sanz et al. 2004). These derivatives had been found only in the defensive secretion of the beetle, *Necrodes surinamensis,* suggesting a potential role of plant defense by these compounds (Sanz et al. 2004).

In our study a significant quantitative variability of these compounds depending on the evolution of flowering (Table 1).

**Table 1:** Concentration of the main components (%) of the essential oils from flowers and leaves of *L. luisieri*

Compound	Leaves				Flowers		
	L1	L2	L3	L4	F2	F3	F4
α-Pinene	1.0	1.4	0.4	0.2	3.4	1.1	–
Trans-α-necrodyl acetate	8.1	18.7	16.9	5.1	20.3	16.6	1.8
Trans-α-necrodol	–	–	–	–	4.1	–	–
β-Pinene	0.9	0.2	0.11		0.1	–	
*p*-Cymene	4.5	–	–	0.2	0.1	1.2	–
*o*-Cymene	–	4.0	1.2	0.4	0.2	0.2	1.2
*m*-Cymene	0.3	0.3	0.3	–	–	0.2	0.4
Camphene	0.1	0.1	0.1	0.2	0.8	0.7	0.2
1,8-Cineole	16.4	13.9	15.5	6.3	2.7	3.5	4.0
Fenchone	–	–	–	1.6	1.3	–	–
Camphor	1.1	1.6	3.1	42.9	20.8	8.2	12.0
Linalool oxide	1.7	1.1	1.5	0.4	0.4	1.2	1.5
Trans-linalool oxide	–	–	1.5	–	–	0.2	–
Linalool	0.9	1.6	1.8	0.3	1.8	2.9	1.4
1,2,3,3-Tetramethylcyclopenten-4-one	0.3	0.5	0.4	0.2	0.1	0.3	0.6
3,4,4-Trimethyl-2-cyclohexanone	–	1.4	–	–	0.5	0.9	2.0
2,3,4,5-Tetramethyl-2-cyclopenten-1-one	–	0.2	0.4	–	–	0.3	0.5
1,1,2,3-Tetramethyl-4-hydroxymethyl-2-cyclopentene	–	3.1	–	–	–	2.0	–
1,2,3,3-Tetramethylcyclopenten-4-one	0.3	0.5	0.4	0.2	0.1	0.3	0.6
Limonene oxide	–	0.2	0.4	0.2	0.6	0.5	0.5
Limonene	–	–	–	–	0.7	0.1	–
T-muurolol	–	0.1	0.9	2.0	–	0.3	–
*p*-Mentha-1,5-dien-8-ol	–	0.5	0.3	0.4	0.2	2.3	1.0
Eucarvone	1.9	–	–	–	–	–	–
Verbenone	1.6	0.5	–	–	0.3	2.0	1.3
3,4,4-Trimethyl-2-cyclohexen-1-one	–	1.4	–	–	0.5	0.9	2.0
Myrtenal	0.4	0.3	0.2	–	–	–	0.3
Cubenol	–	–	1.7	–	0.4	1.1	–
Viridiflorol	4.1	–	–	–	1.0	1.1	6.2
Ledol	–	–	3.7		–	3.9	1.8
Dihydrocarveol acetate	–	2.4	2.6	–	1.9	2.5	2.0
Lavandulyl acetate	7.2	0.8	4.0	–	–	–	–
Neryl acetate	–	–	0.9	–	0.7	1.1	–
Epiglobulol	–	1.7	–	–	–	2.1	2.9
Trans-pinocarveol	0.5	–	–	–	–	–	0.3
α-Cadinene	–	–	–	–	0.3	–	0.7
δ-Cadinene	1.1	0.6	0.5	–	–	1.5	–
τ-Cadinene	–	0.2	–	–	–	–	–
δ-Cadinol	–	–	–	–	3.3	–	0.5
Caryophyllene oxide	0.9	0.8	–	–	0.5	1.3	–
Globulol	1.3	–	–	–	–	–	–
1,1,2,3-Tetramethyl-4-hidroxymethyl-2-cyclopentene	–	3.1	–	–	–	–	–
α-Cyclogeraniol	–	–	2.1	–	–	–	–
p-menth-8-en-2-ol	–	–	–	1.3	–	–	–
Carveol	–	0.1	–	–	0.2	0.3	0.2
Ledene	–	–	–	–	–	–	1.1
Teresantalol	–	–	–	–	1.8	2.3	–
Yield (% V/W)	2.8	1.1	0.7	0.2	2.0	0.8	1.0
Total compounds identified (%)	58.1	69.1	73.1	68.7	79.5	65.3	65.2

*L1* leaves and flowers before flowering, *L2*; *F2* leaves and flowers early flowering, *L3*; *F3* leaves and flowers before flowering, *L4*; *F4* leaves and flowers end of flowering

### Total phenols

The total phenolic (TP) content was examined using the Folin–Ciocalteu’s reagent and expressed in terms of gallic acid (Swain and Hillis 1959). Using Eq. (1) it was obtained a TP value of in mg gallic acid/L methanolic solution, with the mass of extract obtained. The total phenols value it is possible to observe in Table 2.

**Table 2:** Essential oil yield, total phenolic content and antioxidant activity of the extracts of *L. luisieri* flowers and leaves

Solvent	Sample	Yield (%)	Total phenolic content (mg/g)	Total flavonoids (mg/g)	IC_50_	AAI	
Hexane	Flowers	9.7 ± 0.2	461.0 ± 0.1	7.9 ± 0.3	1193.0 ± 0.2	26.2 ± 0.1	VS
	Leaves	7.4 ± 0.3	636.5 ± 0.1	22.7 ± 0.2	1314.7 ± 0.2	14.8 ± 0.1	VS
Methylene chloride	Flowers	6.4 ± 0.6	620.1 ± 0.1	9.3 ± 0.3	1248.9 ± 0.1	25.0 ± 0.1	VS
	Leaves	7.3 ± 0.4	714.2 ± 0.2	8.3 ± 0.2	1296.6 ± 0.1	24.1 ± 0.1	VS
Ethanol	Flowers	2.4 ± 0.4	1326.0 ± 0.1	28.3 ± 0.1	1069.7 ± 0.1	29.2 ± 0.1	VS
	Leaves	3.1 ± 0.1	1778.0 ± 0.1	27.8 ± 0.1	774.0 ± 0.3	40.0 ± 0.1	VS
Essential oil	Flowers	0.7 ± 0.1	1591.1 ± 0.1	33.1 ± 0.2	1323.4 ± 0.2	23.6 ± 0.1	VS
	Leaves	0.8 ± 0.1	1612.5 ± 0.1	25.0 ± 0.1	1210.5 ± 0.2	25.8 ± 0.1	VS

*VS* antioxidant capacity very strong

### Total flavonoids

The concentration of flavonoids of all extractions was determined using spectrophotometric method with aluminum chloride. The content of flavonoids was expressed in terms of quercetin equivalent (Latoui et al. 2012). Flavonoids were presented in small amounts compared to the phenols.

Using Eq. (2) was obtained a total flavonoid (TF) value in mg of quercetin/mL methanolic solution, with the mass of extract obtained. The total phenols value is possible see in Table 2.

### Antioxidant activity

A freshly prepared solution of 2,2-diphenyl-1-picrylhydrazyl (DPPH) has a deep purple color with an absorption maximum at 517 nm. This purple color usually disappears when the antioxidant molecules eliminate free radicals DPPH.

Analyzing fifty samples it was found that the sample with the concentration of 1.500 µg/mL show the higher value of percentage inhibition (%I) which is variable value depended of the extract or oil. The sample with the lowest value %I is a sample with a concentration of 50 µg/mL, the same as going to the higher concentration. The %I values obtained for each concentration construct a linear relationship with the Eq. (3); this equation allowed the calculation of an IC50 value and allowed antioxidant activity index (AAI) value calculated. All results of antioxidant activity are shown in Table 2.

## Antibacterial activity

### Antibacterial activity of diffusion agar plate with cylindrical cavities

The results of antibacterial screening are obtained by measurement of the inhibition halo observed in mm (zone of inhibition) for each different strain. The comparison with weak, moderate and strong inhibition it does following the literature Ostrosky et al. 2008. The inhibition halos for antibiotics, DMSO and essential oil (flowers and leaves) (mm) are represented in Table 3.

**Table 3:** Comparison of inhibition zones obtained by diffusion technique in agar with CMI and CMB of the essential oil of *L. luisieri* and the controls

Essential oil	Strains	Inhibition zone (mm) in cylindrical cavities		Inhibition zone (mm) in vapor diffusion tests	MIC (µg/mL)	MBC (µg/mL)
			Concentration (mg/70 µL)			
Leaves	*Escherichia coli*	17.9 ± 0.3	61.6	31	22,000	22,000
	*Staphylococcus aureus*	SI		40	11,000	11,000
	*Salmonella* spp.	8.9 ± 0.3		0	22,000	22,000
Flowers	*Escherichia coli*	22.3 ± 0.2	61.6	30	22,000	22,000
	*Staphylococcus aureus*	SI		41	22,000	22,000
	*Salmonella* spp.	11.2 ± 0.2		0	22,000	22,000
Gentamicin	*Escherichia coli*	24.2 ± 0.1	0.01	–	–	–
	*Staphylococcus aureus*	29.2 ± 0.1		–	–	–
	*Salmonella* spp.	24.2 ± 0.1		–	–	–
Penicillin G	*Escherichia coli*	0.0 ± 0.0	0.0005	–	–	–
	*Staphylococcus aureus*	48.2 ± 0.1		–	–	–
	*Salmonella* spp.	0.0 ± 0.0			–	–
DMSO	*Escherichia coli*	0.2 ± 0.1	CS	–	–	–
	*Staphylococcus aureus*	0.2 ± 0.1		–	–	–
	*Salmonella spp*	0.2 ± 0.1		–	–	–

*SI* strong inhibition, visual bacterial growth, only at the periphery of the culture medium, *CS* concentrated solution

The diffusion in agar plate the essential oil showed a strong inhibition against *Staphylococcus aureus*, it happens with the flower and leaves oil.

Against *E. coli* the test exhibited a moderate effect of inhibition, but the effect is somewhat higher when we use a flower oil.

Finally, when we tested against wild strain of *Salmonella* spp. are brought a weak/moderate effect of inhibition, but stressing a slightly greater inhibitory effect on oil from the flowers.

Certainly, that the antibacterial activity of this oil can be explained by the presence of a trans-α-necrodyl acetate. The biologic activity of this oil against *S. aureus*, observed in this work coincides with the results obtained by Baldovini et al. (2005).

### Antibacterial activity of vapor diffusion test in agar plate

The vapour diffusion in agar plate has showed interesting results. Having as positive controls the antibiotics Gentamicin (broad spectrum) and Penicillin G (Gram-positive) and comparing the inhibition halos of the essential oil with the antibiotics halos. The inhibition halos (mm) are represented in Table 3.

Analyzing the results obtained with wild strain of *Salmonella* spp., it was found that there was no inhibitory effect with the flowers or leaves oils.

Against *S. aureus* it’s possible detected a strong inhibition, when used the leaves or flowers oil, compared with Gentamicin. However, when we compared the same samples with Penicillin G detects a moderate inhibition.

Finally, when we analyzed the results obtained using *Escherichia coli* was detected that has a strong inhibitory effect, using the leaves or flower oils.

### Method of macrodilution—minimum inhibitory concentration (MIC) and minimum bactericidal concentration (MBC)

The interpretation and criteria for MIC and MBC were the following: the tube corresponds to the highest dilution with no visible turbidity to the naked eye, this is considered the MIC Observed. Therefore, MIC Real will lie between MIC Observed and adjacent lower dilution. For the MBC, it is always considered less than or equal to the MIC (in terms of dilution or concentration of antimicrobial agent), given that it represents the bactericidal effect on bacterial growth on solid medium, corresponding to inhibition of at least 99.9 % the original inoculum. According to the methodology described, we obtained the values summarized in Table 3.

When we observed the results it is possible verify that the values are identical, except for the value using the leaves essential oil against *S. aureus*. When we used this strain with flower and leaves the same doesn’t verify. For more accurate results, we should have done trials with intermediate dilutions in the results of MIC and MBC Reals. So we would have a better interpretation of the results given in MIC and MBC.

### Comparison between inhibition halos and the MIC and CMB

The antibacterial activities, evaluated by vapor diffusion test and diffusion agar plate with cylindrical cavities, demonstrated satisfactory and reliable figures, but we consider that there was no full agreement with the macro- assays for the determination of MIC and CMB, with regard to obtained concentration values (higher MIC and CMB concentrations compared to the diffusion agar plate with cylindrical cavities). That is, with respect to vapor diffusion, cylindrical cavities, MIC and MBC tests in the leaves, we have concordant results for *S. aureus*, thereby obtaining higher inhibition values compared with the other strains. For flowers, there is only agreement in the vapor diffusion and cylindrical cavities tests, anyway, there is greater inhibition *S. aureus*.

In order to verify that the values of MIC and CMB are consistent with the agar diffusion tests performed, it should be held in future studies, intermediate dilutions.

## Conclusions

In summary, the results presented here contribute to the knowledge of chemical composition and antibacterial activity *L. luisieri*.

The common major component for essential oils of *L. luisieri* is trans-alpha-necrodyl acetate. This oils show less variability in their composition compared to previously reported from populations of central and southern Spain rich in 1,8-cineole, fenchone, camphor and 2,3,4,4 tetramethyl-5-methylene-2-cyclopenten-1-one (Sanz et al. 2004; González-Coloma et al. 2006), Southern or the Portuguese population with 1,8-cineol as main compound and a trans-alpha-necrodyl acetate present in lower amounts (Matos et al. 2009), indicating a significant chemical difference. In this work was not always possible to verify that 1,8-cineole is the major compound, only in the beginning of flowering (both in leaves and in flowers) 1,8-cineole is the main component, in all other samples, trans-alpha-necrodyl acetate is the major compound.

The essential oil extracted by hydrodistillation was used to analyze the chemical composition and antimicrobial tests; the essential oil was extracted by Soxhlet was used in the total phenols, total flavonoids and antioxidant activity.

It was observed too that the various extracts have a large amount of phenolic compounds, thus resulting in a high content of phenols and flavonoids.

Due to its high composition of phenolic compounds, their antioxidant activity, it becomes of great interest, since is very high in comparison with other plants.

After analyzing all results is possible to say that the diffusion with cavities and the vapor diffusion show very different results. The most important difference is the inhibitory effect *Salmonella* spp., since in the diffusion agar plate with cylindrical cavities have a weak or moderate inhibitory activity, and in vapor test in agar plate doesn’t have inhibitory activity. The other strains, *E. coli* and *S. aureus*, the inhibitory activity can vary moderate to strong. Anyway, we found that both methods, show that both the leaf and flowers oils, have higher antibacterial activity for *S. aureus* and lower for *Salmonella* spp.

The values of MIC and MBC, allow us to conclude the presence of antibacterial activity of these oils against tested strains. By the results obtained its possible verify that CIM or CBM against *S. aureus* its strong when compared with the *E. coli* or *Salmonella* values.

The antibacterial mode of action of essential oils as reflected in these will lead to the death of the cell (microorganism). This can be suggested by the composition of each essential oil.

It is believed that the antibacterial activity of the essential oils is not assigned to one specific mechanism, but there are various targets in the cell, due to the various existing components in the essential oils. Not all of these mechanisms are performed separately. Some, are affected as a consequence of other mechanisms.

## Methods

### Raw material

 Samples of the aerial parts (leaves and flowers) of *L. luisieri* growing wild in Portugal, Penamacor (40°12′ 06,741″N; 07°06′ 22,085″W) were collected during the flowering phase (before flowering, early flowering, full flowering and end of flowering). At least, ten plants were collected in order to have a composed sample. At the laboratory, the plants were dried during 1 month in dark at room temperature.

### Extraction method

The successive extractions were made with hexane, methylene chloride and ethanol in a Soxhlet apparatus. The extractions were made in triplicate with samples of 20 g of leaves or flowers each. The solutions were cooled and then concentrated at room temperature under vacuum.

The essential oils were prepared by hydrodistillation (100 g of leaves), using a standard apparatus recommended in the European Pharmacopeia during 2 h.

## Chemical analysis of essential oils

### GC

The oils were analyzed on Agilent Technologies 7890A GC-System apparatus equipped with a DB5-MS fused silica capillary column, 30 m × 0.25 mm i.d., film thickness 0.25 µm of polydimethylsiloxane (J&W LTM Column Module). The initial column temperature is 50 °C (5 min), rising from 50 to 270 °C at 7 °C/min, and the final temperature is 250 °C; injector temperature: 270 °C; carrier gas: He (1 mL/min). The injected volume was 1 µL.

The relative concentration was calculated using the Chemstation Software, which allowed the assimilation of the percentages of the peak areas to the percentages of the various constituents and the NIST Mass Spectral Software.

### GC–MS

Samples were analyzed by GC–MS on Agilent Technologies 5975C, Inert XL MSD with Triple-Axis detector using same experimental conditions as described above. The mass spectrometer operating conditions were: ionization voltage, 70 eV; ion source 230 °C.

### Total phenols

Total phenol (TP) concentration was measured by the colorimetric Folin–Ciocalteau assay (Swain and Hillis 1959) using a UV–Vis spectrophotometer, model Evolution 160 (Thermo Fisher Scientific, Madison, USA) at a wavelength of 765 nm. TP concentration was calibrated (R^2^ = 0.997) using standard methanolic solutions of gallic acid (50–500 µg/mL), and expressed as milligrams of gallic acid equivalent per gram of dry biomass (mgGAE/gDB) by means of the linear relationship:

1 $${\text{ABS}} = 0.0011{\text{TP }} - 0.0381$$

Analyses were performed in triplicate.

### Total flavonoids

The total flavonoids (TF) content of the extracts was determined using the colorimetric method described by Latoui et al. (2012) of deionized water, we added 0.075 mL of 5 % sodium nitrite solution; after 5 min of reaction 0.15 mL of 10 % aluminum chloride; after additional 6 min 0.5 mL of 1.0 M NaOH, and finally deionized water into a final volume of 3 mL. The absorbance of the mixture was determined at 415 nm using the same spectrophotometer as above. Analyses were performed in triplicate. The calibration straight line (R^2^ = 0.999) was made using standard methanolic solutions of quercetin in the range 12.5–200.0 µg/mL:

2 $${\text{ABS}}_{415} = 0.0056{\text{TF}} + 0.0072$$

### Antioxidant activity

The antioxidant activity of the extracts was measured in terms of hydrogen-donating or radical-scavenging ability by means of the radical 2,2-diphenyl-1-picrylhydrazyl (DPPH) method Brand-Williams et al. (1995) which is widely used to describe the antiradical power of different matrices (Aliakbarian et al. 2009). For each sample, seven different dilutions ranging from 50.0 to 1500.0 µg/mL in methanol were prepared. For each extract, 0.10 mL of diluted sample was mixed with 3.90 mL of DPPH· methanolic solution (0.10 mM). The reaction mixtures were shaken and incubated for 90 min in the dark at room temperature, and then the absorbance was read at 517 nm using the same spectrophotometer as above. The inhibitory activity was calculated and the IC50 (concentration of the extract able to inhibit 50 %) was calculated graphically with the concentration of the extract versus the corresponding inhibitory effect (% I). The antioxidant activity was expressed as the antioxidant activity index (AAI) Analyses were performed in triplicate (*R* ^2^ = 0.996).

3 $${\text{ABS}}_{517} = 0.0248{\text{DPPH}} + 0.0132$$

## Antibacterial activity

### Characteristics of strains

The microorganism used in these tests were obtained from American Type Culture Collection (ATCC), distributed by Culti-loop^®^ (Oxoid Ltd). The selected strains were *S. aureus* (ATCC 25923), *Escherichia coli* (ATCC 25922) and a wild strain of *Salmonella* spp., obtained from coprocultures in the Clinical Analysis Laboratory Brito Rocha, Lda. The pure cultures of each microorganism tested were preserved in *slant* (prepared in the laboratory) in the Muller-Hinton Agar (MHA).

### Culture medium

To determine the antibacterial activity, we used the following resources and solutions: pre-prepared commercial cards of Muller-Hinton Agar (MH2, Ref 43301, BioMérieux SA) and a Muller-Hinton Broth (MHB, Ref 724245-Oxoid Ltd). The MH2 is already prepared and have thick of 4 mm. MHB preparation was performed according to the manufacturer instructions.

In the broth dilution method by macrodilution technique for determination of MIC and MBC, was used MHB medium supplemented with 5 % DMSO, to allow a better oil solubility to the medium. The subsequent colonies count was done in MH2 medium. To test the effect of vapor of essential oils also was used MH2.

### Standardization of inoculum

The standardization of the inoculum was performed once with the same density affects the results of screening. To ensure the concentration of colonies, based on measuring the turbidity of 0.5 McFarland scale, thereby resulting in a concentration of 10^8^ CFU/mL approximate. The wavelength of 550 nm (T ≈ 74.9 % A ≈ 0125) for such a densimeter DENSIMAT (BioMérieux Code 99234) was used. Subsequently, dilutions and addition of colonies ensured the optimal concentration for the screening of bacterial (Pombal et al. 2014).

### Method of diffusion in agar plate with cylindrical cavities

The antimicrobial activity was evaluated according to the agar diffusion method proposed by the Clinical and Laboratory Standard Institute (CLSI, Wayne, Pennsylvania, PA, USA), but replacing the sterile paper discs impregnated with the sample by cylindrical cavities of 4 mm in height and 5 mm in diameter (CLSI document M02-A10 2009a; Alves et al. 2008).

In this test we used control board with 2 antibiotics (Penicillin G and Gentamicin) and a solvent DMSO. The essential oil was introduced in the swab inoculum (10^6^ CFU/mL) and pressed into the wall of the tube to remove excess liquid. The cylindrical cavities were filled with 70 µL of oil and their controls, after a rest period of 15 min at room temperature, which allows the diffusion of the components, the plates were incubated for 18–24 h/37 ± 0.5 °C.

### Method of the effect of vapor—diffusion in agar plate

To study the antibacterial activity due to volatility (vapor effects) of the leaves and flowers oil of *L*. *luisieri*, we used the method described by Lisin et al. (1999). The crude oil and corresponding fractions were introduced in the swab inoculum (10^6^ CFU/mL) and pressed into the wall of the tube to remove excess liquid. It then spread evenly with the swab, the inoculum on the agar plate MH2 and left to stand for 15 min so they absorb the culture medium around the inoculum. In order to do a comparative test with the agar diffusion method using the technique of cylindrical cavities, it was added the same amount, 70 μL of the sample in the center of the lid of the Petri dish, at a distance of approximately 1 cm from the agar inoculated, inverted the plate and incubated during 18–24 h/37 ± 0.5 °C.

### Method of Macrodilution—Minimum Inhibitory Concentration (MIC) and Minimum Bactericidal Concentration (MBC)

To determine the MIC and MBC the technique of macrodilution in MHB broth was used, according to the methodology of CLSI M07-A8 (2009b).

In four sterile tubes. In first tube was placed 0.5 mL of inoculum 5 × 10^5^ UFC/mL prepared in MHB broth +5 % DMSO and 0.4 mL of MHB broth +5 % DMSO. In the remaining three tubes was placed 0.5 mL of inoculum 5 × 10^5^ UFC/mL (prepared in MHB broth +5 % DMSO). After, in the first tube, was added 0.1 mL of essential oil making a total volume of 1 mL and the tube was homogenized. Then 0.5 mL was withdrawn from the first tube and transferred to the second tube and so on until the four dilution. The positive control is made only by MHB broth +5 % DMSO inoculated. As a negative control, we used 1 mL of the essential oil and 0.4 mL of MHB broth +5 % DMSO. All test tubes, mixed thoroughly, were incubated for 18–24 h/37 ± 0.5 °C.

After incubation, the tubes were visually examined.

For the determination of the MBC, samples were inoculated by spreading 0.1 mL of each tube (including controls) on MH2 agar and incubating during 18–24 h/37 ± 0.5 °C and then proceeding to count the colony forming units (CFU). All assays were performed in triplicate (NCCLS document M26-A 1999).
